# Feasibility and effectiveness of cardiac telerehabilitation for older adults with coronary heart disease: A pilot randomized controlled trial

**DOI:** 10.1016/j.conctc.2024.101365

**Published:** 2024-09-12

**Authors:** Jing Jing Su, Arkers Kwan Ching Wong, Xi-Fei He, Li-ping Zhang, Jie Cheng, Li-Juan Lu, Lan Lan, Zhaozhao Wang, Rose S.Y. Lin, Ladislav Batalik

**Affiliations:** a School of Nursing, Tung Wah College, Hong Kong; b School of Nursing, The Hong Kong Polytechnic University, Hong Kong SAR, China; c Department of Nursing, Tongji Hospital, Tongji Medical College of Huazhong University of Science and Technology, China; d Elaine Hubbard Center for Nursing Research on Aging, School of Nursing, University of Rochester Medical Center, USA; e Department of Rehabilitation, University Hospital Brno, Brno, Czech Republic; f Department of Physiotherapy and Rehabilitation, Faculty of Medicine, Masaryk University, Brno, Czech Republic; g Department of Public Health, Faculty of Medicine, Masaryk University, Brno, Czech Republic

**Keywords:** Cardiac telerehabilitation, Coronary heart disease, Older adults, Pilot, Randomized controlled trial

## Abstract

**Background:**

Cardiac rehabilitation is a beneficial multidisciplinary treatment of exercise promotion, patient education, risk factor management, and psychosocial counseling for people with coronary heart disease (CHD) that is underutilized due to substantial disparities in access, referral, and participation. Empirical studies suggest that cardiac telerehabilitation (CTR) have safety and efficacy comparable to traditional in-person cardiac rehabilitation, however, older adults are under-reported with effectiveness, feasibility, and usability remains unclear.

**Methods:**

The study randomized 43 older adults (84 % males) to the 12-week CTR intervention or standard of care. Guided by Social Cognitive Theory, participants received individualized in-person assessment and e-coaching sessions, followed by CTR usage at home. Data were collected at baseline (T0), six-week (T1), and 12-week (T2).

**Results:**

Participants in the CTR intervention group showed significant improvement in daily steps (T1: β = 4126.58, p = 0.001; T2: β = 5285, p = 0.01) and health-promoting lifestyle profile (T1: β = 23.26, *p* < 0.001; T2: β = 12.18, *p* = 0.008) across study endpoints. Twenty participants completed the intervention, with 40 % used the website for data-uploading or experiential learning, 90 % used the pedometer for tele-monitoring. Improving awareness of rehabilitation and an action focus were considered key facilitators while physical discomforts and difficulties in using the technology were described as the main barriers.

**Conclusions:**

The CTR is feasible, safe and effective in improving physical activity and healthy behaviors in older adults with CHD. Considering the variation in individual cardiovascular risk factors, full-scale RCT with a larger sample is needed to determine the effect of CTR on psychological symptoms, body weight and blood pressure, and quality of life.

## Highlights

• CTR intervention is feasible and acceptable in older adults with CHD.
• This study identified a significant improvement in physical activity and health-promoting lifestyle profile.
• No adverse events occurred during the CTR.

## 1 Introduction

Coronary heart disease (CHD) is well-documented as a common cause of death among individuals aged 60 years and above [1]. Prevalence of CHD increases with age due to the progression of long-established cardiovascular risk factors, such as hypertension, dyslipidemia, and physical inactivity [2,3]. The aging process itself contributes to an elevated risk of CHD through various pathophysiological mechanisms, including heightened arterial stiffness, disrupted blood pressure regulation, increased levels of oxidative stress and inflammation, and age-related subcellular changes [4]. Additionally, older adults are particularly susceptible to deconditioning, atypical symptoms, and non-compliance with prescribed treatments [5]. Managing CHD in older patients is inherently complex due to the frequent coexistence of multiple chronic conditions (known as multimorbidity), the use of multiple medications (referred to as polypharmacy), frailty, and the presence of geriatric syndromes [6]. Consequently, there is a compelling rationale for the implementation of cardiac rehabilitation (CR) programs tailored to the needs of older adults, aiming to address the unique challenges for optimizing health benefits [7].

CR is a multicomponent intervention, including individual assessment, physical activity promotion, health education, psychological counseling and cardiovascular risk management that is individualized to meet the specific needs of patients [5,8,9]. Tailored physical activity promotion are widely used to overcome the chronic sedentariness and offset the effects of hospitalization related deconditioning for older adults [2]. Risk reduction is achieved by providing health assessment that identifies modifiable CVD risk factors such as comorbidities, tobacco, and stress [2,4]. Education and counseling from professionals are incorporated to promote and sustain life-long healthy behaviors and enhance feelings of being supported [10]. The multifaceted nature of CR is crucial for addressing patients' health concerns holistically and achieving a combined effect in managing multiple risk factors. A large trial investigated the effects of CR on older adults with CHD and found that mortality rates were 21 %–34 % lower in patients who utilized CR compared to those who did not, which is consistent with studies conducted in younger populations [11]. However, less than 25 % of eligible older adults participated in one or more CR session and about 5 % completed center-based CR following a cardiac event, due to lack of awareness, difficulties in traveling and adhering to medical appointments, lower referral rates, and experience of pain and fatigue [[12], [13], [14], [15]].

CTR comprises of telemonitoring [16], online patient education [17], and remote guidance/supervision [18], is safe and cost-effective to deliver CR for older adults [[19], [20], [21]]. This approach allows older adults to access CR from the comfort of their homes, overcoming travel difficulties by utilizing smartphones, laptops, and wearable monitoring sensors [22]. Compared to traditional center-based CR, these new delivery methods offer greater ecological validity. For example, telemonitoring of daily steps or heart rate enables continuously health data collection, while healthcare professionals can provide personalized remote feedback to assist individuals in managing their heart condition within the context of their overall health, including comorbidities [2,23]. During counseling, patients could show the CR professionals, for instance, their meals/refrigerator for remote observations and suggestions [24]. Despite the benefits, the non-usage of e-platforms and devices presents fundamental challenges, with reports of high discontinuation rates and low levels of e-health literacy [25]. Negative self-perception of aging and misinformation about the disease can also hinder the adoption of these interventions [26]. Understandably, the intervention usage is at older adults discretion and requires more motivation and input to enhance engagement [27]. Importantly, age itself is not a barrier to technology usage and some Internet/App based intervention have been successfully used in the older adults to improve physical activity [28].

One recent systematic review and meta-analysis suggested the effectiveness of CTR in improving functional capacity, cardiorespiratory fitness and quality of life of older adults [20]. However, none of the included studies focused on older adults with CHD, with average age of the included studies ranged from 54 to 66 years old [20]. Another study showed a significant improvement in cardiorespiratory fitness of peak oxygen uptake among older adults receiving CTR compared to standard of care [21]. However, these studies did not address the feasibility, technology usage among older adults with CHD were missing. Furthermore, the reporting of various health outcome parameters sensitive to CR participation, such as physical activity level, health-promoting lifestyle, psychosocial well-being, and risk factor management, was lacking. Considering the high penetration rate of smartphones in China (72 % as of 2022) [29], the aim of this study was to evaluate the effect, feasibility, and usability of CTR among older adults with CHD.

## 2 Materials and methods

### 2.1 Study design

The study is a pilot two-arm randomized controlled trial with qualitative process evaluation (registration number: ChiCTR1800020411). The study inclines to CONSORT guidelines and extension for reporting pilot randomized controlled trials. The study complied with the Declaration of Helsinki and was approved by the Joint Chinese University of Hong Kong – New Territories East Cluster Clinical Research Ethics Committee (Protocol Record No.2018.469).

### 2.2 Setting and participants

The investigation took place at a tertiary hospital located in Wuhan, China, in the year 2019. Participants were recruited consecutively from cardiovascular department of a tertiary hospital. They were recruited when their CHD were treated with percutaneous coronary intervention and/or medication, and became stable as confirmed by physicians.

The inclusion criteria were [1]: adults aged ≥60 [2], diagnosed with CHD [4], able to communicate and read Chinese [5], owning a smartphone, and [6] having no prescribed physical activity restriction. Patients who had acute psychiatric illness, a life-limiting condition (e.g., cancer), diagnosis of cognitive impairment, absolute and relative contradictions to exercise testing and training according to the American Association of Cardiovascular and Pulmonary Rehabilitation (AACVPR) guideline were excluded.

### 2.3 Sample size estimation

The sample size estimation is based on the primary outcome, which is the physical activity level. We adopted a medium effect size observed in a previous review using CTR for improving physical activity [30]. For intervention with an estimated effect size of medium, a pilot trial sample size of 20 per arm was used and a total sample of 40 were needed for this study [31].

## 3 Cardiac tele-rehabilitation intervention

### 3.1 Framework

The intervention was guided by the Social Cognitive Theory (SCT), with the primary purpose to promote behavior change, especially physical activity [32]. The SCT highlights that to promote behavioral change, a reciprocal causation between individuals’ cognitive (i.e., personal beliefs), behavioral (e.g., self-efficacy) and environmental factors (e.g. professional consultation) should be fostered [32,33]. More specially, the CTR intervention incorporated individual goal-setting cycle, through which the CTR nurse assessed the patient individually, elaborated self-care deficits, and co-developed personal behavioral change goals with action plans. The CR website and professional consultation were provided to support goal attainment by enabling knowledge and skills acquisition. Tele-monitoring was offered to allow real-time data collection for tracking the progress and allowing professional feedback.

### 3.2 Intervention

The intervention began with an in-person assessment conducted by a registered nurse trained in CR before hospital discharge. During which the nurse conducted 6-min walk test to assess their exercise capacity (another cardiac nurse observed patients' Holter monitored ECG at the nurse station during the test) and interviewed the participants to understand their exercise habits, eating habits, stress management practices, smoking status, and social aspects related to disease management. Then, the nurse introduced guideline-based CR recommendations and compared them with participants' usual practice. The gap between the participants reported behaviors and the CR recommendations was highlighted, and the benefits of behavior change on preventing disease progression was elaborated. The nurse supported participants to collaboratively generate behavior change goals and action plans according to their fitness level, habits, guideline recommendations and preferences. The goal setting for physical activity is successive, gradually increasing the frequency, duration, and intensity to achieve at least 150 min of moderate weekly exercise, and slow to brisk walking is the primarily recommended activity type among older adults. Other types of moderate intensity physical activities such as square dancing, Tai Chi, if preferred by participants, were discussed with reference to the American College of Sports Medicine guideline [34]. The nurse taught participants the use of Borg's ratings of perceived exertion to self-monitor moderate intensity (from “fairly light” to “somewhat hard” on a scale of 6–20). The physical activity plan was reviewed and approved by their physicians. In addition, the nurse taught patients about using the CR website for self-learning and pedometer (Mi band) for monitoring daily steps. Lastly, participants were invited to join WeChat (a social media App) chatroom for peer interaction and professional consultation. A booklet with personal goals and action plans, and user manual of the website and pedometer were provided.

The CR website was account and password-protected (accessible via computer/mobile phone). The website design was guided by *Health Literacy Online* [35] to enhance user-friendliness and readability. The content followed the international guidelines and culturally appropriate national recommendations for CR [[36], [37], [38], [39], [40]], including the pathophysiology and manifestation of CHD, physical activity, healthy diet, smoking cessation, stress coping, cardiovascular risk factors management, symptom management and post-PCI management. Since CR adopts a multidisciplinary approach, cardiologists, physiotherapists, nurses, and dietitians with experiences in providing CR services were involved in commenting on the content to ensure its accuracy and appropriateness for CHD patients [41]. The questions and answers for frequently asked questions were posted. All learning content was presented sequentially: i) the role and mechanism, ii) lifestyle changes, iii) actions, and iv) self-monitoring and resolutions to barriers. The website also allowed for self-monitoring that participants were encouraged to upload weekly/daily goal-attainment data (i.e., physical activity, diet checklist, stress level) for graphical visualization and motivational textual feedback.

The nurse moderated peer communication in the WeChat chatroom to build rapport and encourage progress and experience sharing weekly. Meanwhile, patients were encouraged to raise cardiac-related questions consult the CR nurse individually. The nurse seeked professional input from other CR staff if necessary to fully address participants’ questions and evaluate their health status.

### 3.3 Control group

Participants in the control group received standard of care, with a 10-min didactic education on medication usage and lifestyle modification delivered by physicians when delivering discharge summary. They were taught the use of a pedometer for data-collection purposes.

### 3.4 Measurements

Sociodemographic data, including age, education, sex, marital status, employment condition, and co-residency, were collected. Clinical data were retrieved from the medical record, including diagnosis, treatment, and comorbidities.

### 3.5 Primary outcome - physical activity

Physical activity was measured objectively using a pedometer (Mi Band). Subjective measurement was conducted using the International Physical Activity Questionnaire (IPAQ). The IPAQ has demonstrated good reliability for Chinese older adults, with intra-class correlation coefficients ranging from 0.81 to 0.89 for each sub-domain [42].

### 3.6 Other outcomes

The study utilized several validated measurement tools to assess various aspects of the participants' health and well-being. The Health-Promoting Lifestyle Profile II (HPLP-II) was used to measure the participants' healthy behaviors [43], with a Cronbach's α coefficient of 0.67–0.88 reported for the Chinese older adults [44]. Self-efficacy was measured using the Cardiac Self-efficacy Scale (CSES), that evaluates participants' confidence in maintaining function and controlling symptoms [45]. The CSES has demonstrated excellent internal consistency, with Cronbach's alpha values of 0.926 for the Chinese version [46]. Quality of life was measured by the MacNew Heart Disease (MacNew HRQoL), which evaluates the influence of CHD on participants' physical, emotional, and social wellbeing [47]. The MacNew questionnaire has been translated and validated among the Chinese CHD population, with intraclass correlation coefficients ranging from 0.88 to 0.93 [48]. Psychological symptoms were measured by the 21-item Depression Anxiety Stress Scale 21 (DASS-21) [49,50]. The Chinese DASS-21 had Cronbach's alpha≥0.80 [51]. The cardiac physiological risk parameters of body mass index (BMI) (Xiheng, RGZ-120-RT, China), blood pressure (Omron HEM-7124, Japan), and waist circumference were measured.

Lastly, individual qualitative interviews were conducted via video calls with intervention participants who expressed a willingness to share their experiences. These interviews aimed to explore the participants' perceived facilitators and barriers regarding the usage of the intervention.

### 3.7 Data collection

To assess eligibility, a CR nurse reviewed participants' medical records, conducted interviews, and sought guidance from a senior CR nurse in cases of uncertainty. Potential participants were provided with an information sheet and received a verbal explanation of the study from the nurse. They were assured of their right to withdraw from the study at any time without facing any negative consequences. The nurse obtained final confirmation of eligibility from on-site physicians for those patients who agreed to participate. Baseline data collection was performed by two trained research assistants after obtaining written informed consent from the participants in the hospital. Block randomization was employed, using random block sizes generated by the "Random Allocation Software," to assign participants to their respective groups. The group assignments were written down and sealed in opaque envelopes in the predetermined sequence. Another research assistant opened the envelopes and revealed the group assignments to the participants after the baseline data collection. Post-test data were collected at 6 weeks (T1) and 12 weeks (T2) in the middle/after the intervention by the two research assistants, who were unaware of the participants' group assignments and had no prior knowledge about them. These data collection procedures and ethical considerations were implemented to ensure the integrity of the study and protect the rights and well-being of the participants.

### 3.8 Statistical analysis

The data analysis for this study was conducted using SPSS version 28. Descriptive statistics were computed to summarize the characteristics of the study participants. To assess between-group comparability in demographic, clinical, and outcome variables at baseline, the *t*-test, Chi-square test, and Mann-Whitney test were utilized. The generalized estimating equation (GEE) model was employed to analyze changes in the outcome variables between the two groups across the study endpoints. The GEE model follows the intention-to-treat principle, allowing for mathematical handling of missing data and accommodating the group∗time interaction effect, which leads to better estimation of the intervention effect. Sociodemographic and clinical variables that showed between-group differences with p < 0.20 at baseline were considered covariates and adjusted for in the GEE model. Bivariate correlation analysis was conducted to examine the correlation coefficient between the improvement in primary outcomes from baseline to post-intervention and the usage of the intervention. Effect sizes, specifically Hedges' g, were calculated for all mean differences at the 12-week post-intervention (T2). All statistical tests were two-sided, with a significance level of 0.05.

Qualitative content analysis was utilized to provide more explicit description of participants’ experiences. The interviews were audio recorded and transcribed verbatim. The researchers immersed in the data by reading and rereading the transcripts to understand the interview holistically and to identify the meaningful words, phrases, or sentences. Coding was conducted as a cyclical process. Then, the research team discussed the identified codes to reach a consensus. Conceptually related codes will be sorted into sub-categories. The team searched for structures and patterns to connect the subcategories to generate categories. Consensus over coding and categories generation were achieved through ongoing discussion.

### 3.9 Feasibility and acceptability

The objective of this pilot study was to examine the feasibility, acceptability, and clinical effect of a CTR approach for older adults with CHD. Feasibility was defined as the practicality of implementing the individualized in-person assessment and e-coaching procedures and offering different types and levels of support to improve healthy behaviors for health benefits. Specific criteria were set a priori to evaluate feasibility and acceptability, as follows:

Feasibility and usability. Referring to previous review on Internet-delivered interventions to increase physical activity, a study would be considered feasible if (a) withdrawal rate is less than 30 %, (b) recruitment is more than 20 % [48]. The intervention is useable (a) more than 60 % of participants reported high adherence, and (b) more than 75 % of participants utilized technological features [52]. An appropriate indicator of clinical importance for a pilot study is the effect size (ES) [53]. The interventions were deemed clinically important if a minimum effect size of 0.2 was observed in relation to the primary outcome, which in this case was physical activity [54].

## 4 Results

### 4.1 Participant recruitment

**Fig. 1:**
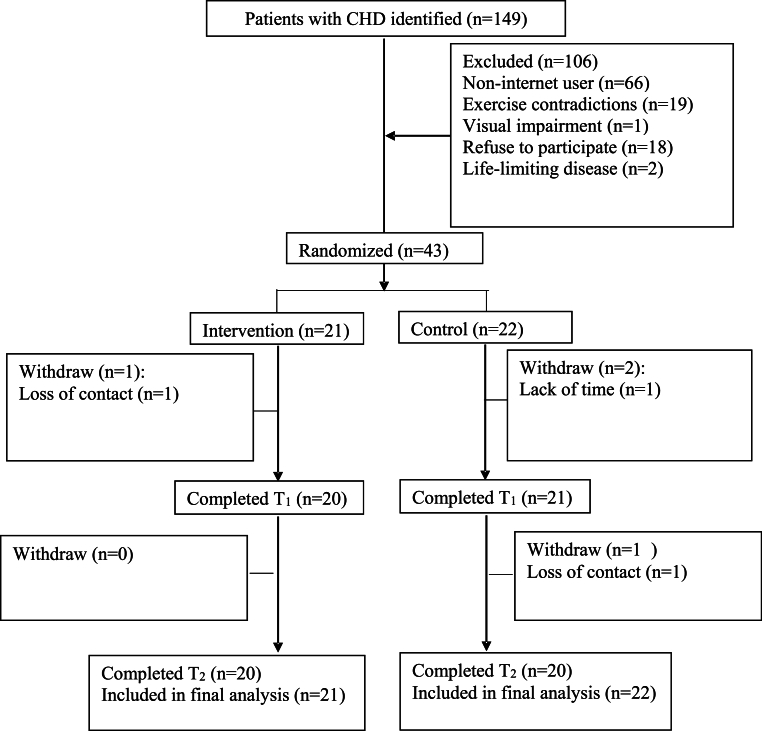
CONSORT patient flow diagram.

A total of 149 participants were reviewed with 43 randomized (21 in the CTR intervention and 22 in the control group). The most common reason for non-eligibility was a Non-internet user (62 %). The CONSORT flow diagram is presented in Fig. 1. The total recruitment rate was 28.9 % and withrawal rate 7 % and satisfying the feasibility limit.

Participants had an average age of 63.49 (±3.34) with average education years of 10 (±3.21). Majority of the participants were male (83.7 %, n = 36), married (97.7 %, n = 42), co-reside with their spouse or family (90.7 %, n = 39). All participants comorbid with at least one chronic condition (e.g., hypertension, diabetes). The baseline characteristics and outcome parameters of participants are presented in Table 1 and no significant differences between groups were detected at baseline. Two sociodemographic and clinical variables (education years and presence of hypertension) with baseline difference *p* < 0.2 were adjusted in the GEE model.

**Table 1:** Baseline data of demographic and clinical characteristics and outcome variables.

Variables	Intervention (n = 21)	Control (n = 22)	*p*
Age (Mean)^a^	63.38 ± 2.96	63.59 ± 3.74	*0.84*
Sex [frequency (%)]^b^			
Male	19 (90.5 %)	17 (77.3 %)	
Female	2 (9.5 %)	5 (22.7 %)	*0.41*
Education years (Mean)^a^	10.81 ± 3.57	8.95 ± 2.90	*0.07*
Marital status [frequency (%)]^b^			
Married	20 (95.2 %)	22 (100 %)	*0.49*
Single/divorced/widowed	1 (4.8 %)	0	
Living status [frequency (%)]^b^			
Alone	3	1	
With spouse	11	10	
With family	7	11	*0.38*
Employment [frequency (%)]^b^			
Retried	13 (61.9 %)	13 (59.1 %)	
Part-time	5 (23.8 %)	7 (31.8 %)	
Full-time	3(14.3 %)	2 (9.1 %)	*0.77*
Smoking [frequency (%)]^b^			
Yes	9 (45 %)	8 (38.1 %)	
Quitted	4 (20 %)	2 (9.5 %)	
No	7 (35 %)	11 (52.4 %)	*0.45*
Drinking [frequency (%)]^b^			
Yes	6 (28.6 %)	15 (68.2 %)	
No	15 (71.4 %)	7 (31.8 %)	*1*
Treatment [frequency (%)]^b^			
PCI	16 (76.2 %)	15 (71.4 %)	
Medication only	5 (23.8 %)	6 (28.6 %)	*0.5*
Hypertension [frequency (%)]^b^			
Yes	17 (81.0 %)	13 (61.9 %)	
No	4 (19.0 %)	8 (38.1 %)	*0.15*
Diabetes [frequency (%)]^b^			
Yes	7 (33.3 %)	4 (21.1 %)	
No	14 (66.7 %)	15 (78.9 %)	*0.31*
Dyslipidemia [frequency (%)]^b^			
Yes	13 (61.9 %)	11 (52.4 %)	
No	8 (38.1 %)	10 (47.6 %)	*0.38*
International Physical Activity Questionnaire			
Sitting (min/week) (Mean)^a^	2191.43 ± 840.13	2502.27 ± 837.02	0.23
Total (vigorous moderate walking exercise) [Median (IQR)]^c^	467.40 (512.98)	490.84 (580.84)	0.89
Daily steps (Mean)^a^	4117.84 ± 3220.81	3041.59 ± 1809.55	0.32
Health Promoting Lifestyle Profile (Mean)^a^	84.43 ± 8.91	82.86 ± 10.88	0.61
Cardiac Self-efficacy (Mean)^a^	3.01 ± 0.77	2.76 ± 0.93	0.33
MacNew Health-Related Quality of Life (Mean)^a^			
Global	5.46 ± 0.56	5.20 ± 0.67	0.17
Physical	5.26 ± 0.64	5.02 ± 0.63	0.21
Emotional	5.55 ± 0.63	5.26 ± 0.79	0.20
Social	5.42 ± 0.59	5.22 ± 0.67	0.31
Depression Anxiety Stress-21 (Mean)^a^			
Total	4.14 ± 4.57	6.81 ± 7.42	0.17
Stress	1.95 ± 2.58	3.81 ± 3.77	0.07
Anxiety	1.62 ± 1.99	1.72 ± 2.12	0.86
Depression	0.57 ± 0.98	1.27 ± 2.16	0.18
Blood pressure (mmHg) [Mean]^a^			
Systolic blood pressure	128.81 ± 18.63	122.00 ± 14.08	0.19
Diastolic blood pressure	74.95 ± 11.09	72.90 ± 10.60	0.55
Body mass index [Mean]^a^	24.72 ± 2.45	25.27 ± 3.05	0.54
Waist circumference (cm) [Mean]^a^	93.00 ± 7.95	89.47 ± 10.25	0.29

Note.

a *t*-test for between-group comparison.

b chi-square for between-group comparison.

c Mann-Whitney test for between-group comparison.

### 4.2 Effects on physical activity

The changes from baseline to each post-test endpoint across outcome variables of both groups are presented in Table 2. Based on the GEE analysis, participants in the intervention group showed a significant group∗time interaction effect in physical activity as measured by daily steps (T_1_: β = 4126.58, *p* = 0.001; T_2_: β = 5285, *p* = 0.01), compared to the control group at 6 weeks and 12 weeks post-intervention. Improvement in physical activity regarding sitting minutes (β = −246.29, *p* = 0.46) and Moderate to Vigorous Physical Activity MET-min/wk (β = 409.34, *p* = 0.29) were not significant at six-weeks. Participants in the intervention group improvement over the control group in physical activity as measured by weekly sitting minutes (β = −879.07, *p* = 0.01), Moderate to Vigorous Physical Activity MET-min/wk (β = 2981.55, *p* = 0.03) at 12 weeks. The effect sizes for physical activity were considered large (Hedge's g = 0.95 to 1.34).

**Table 2:** Generalized estimating equation analysis for the comparison of outcome variables between intervention and control group.

Outcome variables	Time point (IC, CG)	At each post-test time point		Group^a^time effect		Hedges'g
		IG Mean	CG Mean	β (95 % CI)	*p* value	
Daily steps	T1 (n = 15, n = 7)	9717.66 ± 3186.54	5152.03 ± 3943.92	3929.7 (1477.90, 6381.51)	0.002	1.33
Daily steps	T2 (n = 15, n = 5)	10847.70 ± 4558.51	5175.14 ± 2833.09	5285.59 (1216.78, 9354.4)	0.01	1.34
International Physical Activity Questionnaire MET-min/wk						
Sitting minutes	T1 (n = 15,n = 19)	1344.0 ± 669.31	1834.74 ± 604.09	−246.29 (−896.75, 404.18)	0.46	
	T2(n = 12,n = 16)	1200.0 ± 621.96	2197.5 ± 1094.76	−879.07 (−1583.53, −174.60)	0.01	1.08
VMW exercise	T1 (n = 15, n = 19)	2181.0 ± 1197.28	1799.68 ± 972.94	409.34 (−351.41, 1170.20)	0.291	
	T2 (n = 12, n = 16)	5248.25 ± 4736.79	2063.29 ± 1741.77	2981.55 (351.05, 5612.06)	0.03	0.95
HPLP-total	T1(n = 15,n = 19)	109.67 ± 20.77	83.68 ± 11.81	23.26 (10.65, 35.87)	<0.001	1.59
	T2 (n = 12, n = 15)	109.75 ± 11.47	95.87 ± 11.36	12.18 (3.14, 21.21)	0.008	1.22
Self-efficacy	T1(n = 15,n = 19)	3.22 ± 0.42	3.13 ± 0.42	0.16 (−0.54, 0.86)	0.66	
	T2 (n = 12, n = 15)	3.24 ± 0.51	2.84 ± 0.76	−0.25 (−0.8, 0.3)	0.38	
MacNew global	T1(n = 14,n = 19)	6.10 ± 0.40	6.02 ± 0.48	−0.32 (−0.68,0.05)	0.09	
	T2 (n = 12,n = 15)	5.87 ± 0.44	5.67 ± 0.48	−0.06 (−0.58, 0.45)	0.81	
Physical	T1(n = 15,n = 19)	6.25 ± 0.51	6.28 ± 0.53	−0.36 (−0.78,0.06)	0.09	
	T2 (n = 12,n = 15)	5.92 ± 0.44	5.79 ± 0.56	−0.11 (−0.62, 0.40)	0.66	
Emotional	T1(n = 14,n = 19)	5.94 ± 0.42	5.75 ± 0.54	−0.28 (−0.68, 0.13)	0.18	
	T2 (n = 12,n = 15)	5.81 ± 0.46	5.54 ± 0.59	−0.05 (−0.64, 0.66)	0.87	
Social	T1(n = 14,n = 19)	6.19 ± 0.51	6.25 ± 0.60	−0.41 (−0.84,0.02)	0.06	
	T2 (n = 12,n = 15)	5.87 ± 0.51	5.70 ± 0.66	−0.02 (−0.61, 0.57)	0.95	
DASS	T1(n = 15,n = 19)	1.2 ± 2.04	2.52 ± 3.58	1.72 (−1.92, 5.36)	0.36	
	T2 (n = 12,n = 15)	2.58 ± 3.39	3.07 ± 3.45	2.23 (−2.22, 6.67)	0.33	
Depression		0.33 ± 0.62	0.68 ± 1.11	0.68 (−1.05, 2.4)	0.44	
	T2 (n = 12,n = 15)	1.0 ± 1.85	1.13 ± 1.50	0.49 (−0.59, 1.57)	0.80	
Anxiety		0.27 ± 0.46	0.47 ± 0.70	−0.08 (−1.18, 1.02)	0.88	
	T2 (n = 12,n = 15)	0.5 ± 1.0	0.53 ± 0.74	0.05 (−1.22, 1.32)	0.94	
Stress		0.6 ± 1.29	1.37 ± 2.19	1.54 (−0.64, 3.72)	0.17	
	T2 (n = 12,n = 15)	1.08 ± 1.2	1.4 ± 1.91	1.32 (−0.67, 3.31)	0.19	
Weight	T1 (n = 13,n = 14)	69.0 ± 6.21	69.96 ± 9.44	−0.58 (−2.46, 1.30)	0.16	
	T1 (n = 9, n = 11)	66.62 ± 8.21	69.32 ± 10.19	−2.33 (−0.84, 3.77)	0.45	
WC	T1 (n = 8,n = 8)	91.38 ± 8.14	90.38 ± 7.96	−0.21 (−1.73, 1.32)	0.79	
	T1 (n = 2, n = 3)	95.5 ± 10.61	94.0 ± 14.0	−1.48 (−3.70, 0.74)	0.19	
SBP	T1 (n = 9, n = 14)	124.56 ± 13.54	124.5 ± 10.95	−8.17 (−21.92, 5.57)	0.24	
	T2 (n = 7, n = 9)	121.0 ± 16.19	123.11 ± 12.41	−7.85 (−20.94,5.25)	0.24	
DBP	T1 (n = 9, n = 14)	75.67 ± 12.61	77.14 ± 13.55	−2.19 (−12.92, 8.54)	0.69	
	T2 (n = 7, n = 9)	77.57 ± 20.31	73.89 ± 12.54	2.48 (−7.88, 12.84)	0.64	

Note: n = frequency; IG = intervention group; CG = control group; CI = confidence interval; T_1_ = 6 weeks after the intervention; T2 = 12 weeks after the intervention; VMW = vigorous, moderate, walking; HPLP = health-promoting lifestyle profile; DASS = Depress, Anxiety, and Stress Scale; SBP = systolic blood pressure; DBP = diastolic blood pressure; BMI = body mass index; WC = waist circumference. Adjusted for treatment and dyslipidemia.

∗∗p < 0.01.

∗∗∗p < 0.001.

a p < 0.05.

### 4.3 Effects on other outcomes

Participants in the CTR group also showed a significant effect in health-promoting lifestyle profile from baseline to 6 weeks (β = 23.26, *p* < 0.001) and to 12 weeks (β = 12.18, *p* = 0.008) compared with the control group. The improvement in self-efficacy were not significant at both 6 weeks and 12 weeks when compared with the control group. For HRQoL, no significant difference was observed on global HRQoL across study endpoints (T_1_: β = −0.32, *p* = 0.09; T_2_: β = −0.06, *p* = 0.81) between the two groups. There was no significant effect on physical domain (T_1_: β = −0.36, *p* = 0.09; T_2_: β = −0.11, *p* = 0.66), emotional domain (T_1_: β = - 0.28, *p* = 0.18; T_2_: β = −0.05, *p* = 0.87), and social domain (T_1_: β = −0.41, *p* = 0.06; T_2_: β = −0.02, *p* = 0.95) of HRQoL.

Pertaining psychological wellbeing, the intervention group showed no significant improvement in DASS total score (T_1_: β = 1.72, *p* = 0.36; T_2_: β = 2.23, *p* = 0.33) and depression, anxiety, stress subscales compared to the control group.

There is no significant improvement in body weight (T_1_: β = −0.58, *p* = 0.16; T_2_: β = −2.33, *p* = 0.45), waist circumference (T_1_: β = −0.21, *p* = 0.79; T_2_: β = −1.48, *p* = 0.19) when comparing intervention group with the control group. For blood pressure, participants in the intervention group showed no significant differences in systolic blood pressure (T1: β = −8.17, p = 0.24; T2: β = −7.85, p = 0.24) and diastolic blood pressure (T1: β = −2.19, p = 0.69; T2: β = 2.48, p = 0.64) when compared to the control group.

### 4.4 Adverse events

During the study, there were no reported adverse events related to study participation, indicating that the intervention was generally safe for the participants. However, it is worth noting that one participant from the intervention group and one from the control group reported cardiovascular-related re-hospitalization during the study period.

### 4.5 Intervention usage

Regarding intervention usage, all participants attended the in-person assessment and orientation session. Among the 20 participants in the intervention group who completed the study, 100 % utilized one or more of the features provided by the CTR program. Specifically, 40 % of participants (n = 8) used the program's website for activities such as data uploading or experiential learning. Additionally, 90 % of participants used the pedometer for tele-monitoring their physical activity, 100 % participated in the nurse-moderated chatroom, and the 75 % send messages in the chatroom. These results satisfying specific criteria for the intervention acceptability.

The average number of website visits among participants who visited the website was 3.7 (±7.38) and active interaction in an online chatroom 6.9 (±10.08) (messages on the same topic were counted once). The proactive use of the website by participants was found to have a significant positive correlation (correlation coefficient 0.59, p = 0.2) with the improvement in daily steps from baseline to the 12-week post-intervention assessment (T2). However, no significant correlation was found between online chatroom usage and the improvement in daily steps.

Perceived facilitators and barriers for CTR are shown in the Supplementary Table 1.

## 5 Discussion

The present study addressed the limited empirical evidence regarding the effectiveness and feasibility of CTR in older adults with CHD. The main findings indicated that CTR was feasible, safe and effective in improving the physical activity levels and health-promoting lifestyle profiles of older adults. Regarding usability data, tele-monitoring and social media consultations were widely accepted and more frequently used by the participants. However, less than half of the older adults utilized the CR website at home. Interestingly, the frequency of proactive website usage was significantly correlated with improvements in daily steps. This suggests that active engagement with the website positively influenced participants' physical activity levels. Qualitative feedback from participants highlighted the importance of individual assessments to identify personal cardiovascular risk factors and receiving clear and actionable consultations to guide behavior change at home [55]. Older adults reported experiencing physical discomforts and difficulties in learning website usage and self-care skills as the main barriers to their participation in CTR. The study's findings suggest that older adults with CHD should be referred to CTR programs for disease management and rehabilitation. However, to minimize disparities in technology-based interventions, it is crucial to modify the intervention to fit their living environment, personal habits, and technological preferences. This adaptation would enhance the accessibility and usability of CTR programs for older adults, enabling them to fully engage with the interventions and derive maximum benefit.

The study's findings further confirmed the effectiveness of CTR in improving physical activity level of older adults. The perceived barriers of participants in engage physical activity indicate the importance of tailoring physical activity promotion for older adults based on their health condition and functional capacity. The study highlights the notion that any amount of physical activity is better than being sedentary, even if health conditions prevent older adults from achieving the recommended goals. Therefore, CTR programs for older adults should take into account their individual capabilities and limitations. This can be achieved by setting achievable activity goals that are agreed upon by the patients themselves. It is important to involve professionals in identifying barriers and offering solutions to overcome them. Additionally, older adults post CHD may experience limitations in performing some types of physical activity, making light-moderate physical activity such as walking especially vital to meet recommendations [56].

Furthermore, this study observed comprehensive healthy behavior modifications beyond physical activity, indicating that the CTR program had a broad impact on participants' lifestyle changes. Improvement in a broader range of behavioral changes may be reflective of the comprehensiveness of the CR program with an extensive integration of behavior change techniques [57]. Despite the improvement in lifestyle, the CTR intervention showed no significant improvement in anthropometric parameters (i.e., body weight and waist circumference) and blood pressure. This may be because participants had different cardiovascular risk factors; for example, some may not have hypertension or overweight. Another explanation could be related to the challenges faced by older adults in traveling back to the study setting for the collection of hard outcomes such as anthropometric measurements and blood pressure readings. This could have resulted in incomplete or data collection, potentially impacting the statistical significance of the observed changes.

The study captured patients' engagement with the CTR intervention by tracking website visits, data uploading activities, and chatroom interactions. However, the study reached an undesirable intervention usage rate based on the definition of acceptability from a previous review, which considered once per week as an acceptable frequency of engagement [58]. It is important to note that this study recruited relatively young older adults with a high level of education. This demographic characteristic might have influenced their familiarity and comfort with technology-based interventions; it is assumed that older adults with lower education levels or less technological experience may have even lower engagement. However, it is recognized that older adults, regardless of their educational background or technological experience, are often willing to overcome difficulties in learning new things and adopting technologies when they perceive them as beneficial [26]. In the context of CTR, where health education is an essential component for older adults with co-morbidities and multiple medications, it is crucial to make the intervention inclusive to older adults with lower digital literacy levels. To address this, future studies could leverage the CR website as a source for healthcare professionals to initiate health dialogues with patients. This approach would allow professionals to deliver patient education in an interactive and specific way, avoiding frustration associated with technology and self-directed learning among older adults with CHD. By incorporating personalized interactions and guidance, healthcare professionals can bridge the gap between technology and older adults with varying levels of digital literacy, ensuring that they receive the necessary education and support for their CR.

### 5.1 Limitations

Several limitations that should be acknowledged in this study. Firstly, the generalizability of the findings is limited due to the recruitment of older adults who were relatively young, had a higher level of education, and prior internet use experiences. This may not accurately represent the broader population of older adults with CHD, especially considering that the percentage of females with CHD increases with advancing age. Therefore, caution should be exercised when generalizing the findings to older adults with diverse demographics and technological backgrounds. Secondly, the study's non-use attrition, can pose challenges in establishing a cause-effect relationship. The undesirable intervention usage rate may impact the interpretation of the results and the ability to draw firm conclusions about the effectiveness of the intervention. Lastly, the qualitative process evaluation, which aimed to explore participants' perceived facilitators and barriers, had a limited sample size. The inclusion of only a few participants raises questions about data saturation.

## 6 Conclusions

The study demonstrates that a 12-week CTR program can lead to significant improvements in physical activity and health-promoting lifestyle profiles for older adults with CHD who are recovering at home following a cardiac event. The results highlight the usability and feasibility of such interventions, emphasizing the importance of providing ongoing professional support, accommodating user preferences, and enhancing the perceived usefulness and benefits to engage older adults effectively. Full RCT with longer follow-up period is needed to confirm the effectiveness and sustainability.

## Funding

Supported by the 10.13039/501100003243Ministry of Health of the Czech Republic (University Hospital Brno, 65269705).

## CRediT authorship contribution statement

**Jing Jing Su:** Writing – original draft, Methodology, Investigation, Conceptualization. **Arkers Kwan Ching Wong:** Writing – review & editing, Methodology, Formal analysis. **Xi-Fei He:** Writing – review & editing, Project administration, Investigation. **Li-ping Zhang:** Writing – review & editing, Project administration, Investigation. **Jie Cheng:** Writing – review & editing, Project administration, Investigation. **Li-Juan Lu:** Writing – review & editing, Project administration, Investigation. **Lan Lan:** Writing – review & editing, Project administration, Investigation. **Zhaozhao Wang:** Writing – review & editing, Project administration, Investigation. **Rose S.Y. Lin:** Writing – review & editing, Visualization, Validation. **Ladislav Batalik:** Writing – review & editing, Supervision, Methodology, Data curation.

## Declaration of competing interest

The authors declare that they have no known competing financial interests or personal relationships that could have appeared to influence the work reported in this paper.
