# Supplementary material for: Feasibility and effectiveness of cardiac telerehabilitation for older adults with coronary heart disease: A pilot randomized controlled trial
Source: Contemp Clin Trials Commun. 2024 Sep 12;42:101365. doi: 10.1016/j.conctc.2024.101365 (PMC11421251; doi:10.1016/j.conctc.2024.101365)
Supplement: Multimedia component 2 [file mmc2.docx]

Supplementary Table 1 Perceived facilitators and barriers for CTR

| **Perceived facilitators for CTR** |
| --- |
| Altering awareness  Seven participants were interviewed and all expressed that their awareness towards CHD management underwent a significant change through their participation in the CTR program. They acknowledged having misconceptions about CHD prior to the intervention, particularly regarding their individual risks and self-care deficits. The CTR program helped them alter their awareness and understanding of the disease.  *The individual assessment is crucial. My lifestyle was sedentary. (I liked) fishing, chess games, (and playing) musical instruments. I needed more exercise. (Male 65yrs)*  *I was very aware of my goals. I felt my heart rate had increased (during physical activity). I felt OK. Then I increased activity time every other day. (Male 68yrs)*  *I learned from consulting the nurse (online) that the anticoagulant, cholesterol control, anti-hypertensive medicines are important for this illness. (Male, 60yrs)*  *This is the third time I had surgery (PCI). Previously, a nurse told me, do suitable exercise, eat light food, no smoke. But what is suitable? And light? I thought it’s just their job to say those things to everyone. Only from this rehabilitation program, I know (pause) how to exercise, read my steps, from (consulting) the nurse. (Male 62yrs)* |
| Focusing on action  Participants expressed their appreciation for the individualized, clear, and actionable plans provided through the CTR program to guide their behavior changes. They emphasized the importance of co-creating goals and action plans that were tailored to their daily lives. The availability of tele-monitoring and tele-consultation options also played a significant role in helping them form heart-healthy habits and address any concerns they had.  *In the beginning (of the intervention), I walked more than one hour every morning to achieve about 7,000 steps. Recently, I walk a similar time to achieve 9,000 steps every morning. (63, female)*  *The program helped me to form an action plan to make sure that I do not miss my medication. I take it after breakfast and dinner. When I prepare to eat. I get myself a cup of water. I put my medication nearby. When I finish eating. I take my medicine. (Male, 61yrs)*  *The WeChat consultation is very convenient. I have paid attention to eating more vegetables according to my goals. But for several days, I had difficulty in passing stool. Later I consulted the (CTR) nurse, and I realized that eating vegetables alone is not enough. I also need to exercise to promote bowel digestion. (male, 60yrs).* |
| **Perceived barriers for CTR** |
| Participants also shared their experiences of physical discomfort and limitations that hindered their engagement in behavior change  *I have chest pain every four or five days. I was shaking, sweating. I could not speak. It was so painful. I felt I was almost over (pause). I had myself checked by very experienced physicians. But nothing wrong. I wanted to exercise more. But I could not. I do not know when the pain comes and goes. I am trying to manage my quick temper. But the pain makes it difficult.* *(Male, 60yrs)*  *I often felt tired. I tried to walk for half an hour every day. I eat vegetables and a little bit of fish and chicken. I make sure I have enough sleep and rest. But I feel low energy. I followed the advice. I felt it did not work out for me. Even I have been careful to take care of myself. (Male, 61yrs)* |
| Participants expressed difficulties in learning new things from the CTR website and implementing those learnings at home. They also mentioned challenges in using the website itself.  *I watch out my daily steps. But I dislike those buttons to submit my data. (P1059, 64, male, ranked middle)*  *I tried the breathing relaxation (by following) video of your cardiac rehabilitation website. But I am getting old, and trying new things may not be my hobby. I preferred to walk every day with my wife’s accompany. It relaxes me. (P1003, male, 60yrs, ranked low).* |
